# Progress in Skin Diseases

**Published:** 1902-08-09

**Authors:** 


### Progress in Skin Diseases.

(*Continued from page* 311.)

**Light and X-ray Treatment.**—Cases of lupus and rodent ulcer completely cured by X-rays have been reported by D. Morgan. Lewis Jones also obtained almost complete healing of the ulcerated surface of a cancer of the breast by 17 applications of the rays. Kjeldsen [10] has invented a lamp in which the vapour of mercury is made to evolve a light extremely rich in chemical rays, and therefore possessing great bactericidal power. Bang uses iron electrodes with similar results, and claims that his lamp, which only costs about £3, has an effect on bacteria sixty times as strong as the ordinary arc lamp. Hugh Walsham [11] has investigated the properties of the ultra-violet rays which are known to have greater bactericidal power than those of the other end of the spectrum. Their penetrative power is small, and a film of gelatine will stop them entirely, but a block of ice offers hardly any resistance to these rays, though opaque to the red and ultra-red. In using high frequency currents as a source of these rays it was found that an arc between iron points was the most effective source, and that compression of the tissues by a block of ice was advisable to get rid of the blood which is opaque to these rays. He is at present comparing the results of these rays on lupus and other skin diseases with those obtained from the X-ray apparatus. Some, indeed, claim that the power of the latter depends on the secondary production of rays akin to the ultra-violet ones.

**Lupus.**—A. Jamieson [12] showed a case where the features of L. vulgaris and erythematosus appeared on the same patch. There was a ring of nodules, and in the centre a spot covered with splintery scales which showed the usual epidermic tags on the under surface typical of L. erythematosus. He also brought forward an instance of the cure of the latter disease by quinine. Five grains were given three times a day for a year and a half with complete success, though the disease had lasted previously more than 16 years. Poor [13] thinks that the occurrence of transitional forms between the two species of lupus is due to the injury of the tissues by L. erythematosus. The tubercle bacillus may gain a footing in this damaged area, and thus the two diseases may be combined. He does not believe that the erythematous lesion is produced either by the bacilli or their toxins, or that it occurs especially in persons having a tubercular history. The presence of giant cells again is no proof of such an origin, and the tuberculin reaction in these cases does not occur regularly. Hence he thinks the disease has a non-tubercular origin. The results of the light treatment of lupus may be summarised as follows: Sequeira claims 18·5 per cent. of complete recoveries, Malcolm Morris 22 per cent. Sabouraud and Noiré 25 per cent., while Finsen finds that nearly 27 per cent. have remained well from one to five years after the end of treatment. C. H. Gunson [14] advocates treatment by daily spraying with hydrogen peroxide, which leads to a rapid healing of the ulcers and the formation of a healthy, firm cicatrix. In some cases he employs a preliminary scraping, especially when the granulations are excessive. Hallopeau [15] records successes under treatment by permanganate compresses which causes the more rapid healing of ulcerated patches than any other method. Cases which are ulcerated and too extensive for the light treatment are specially adapted for this plan. The compresses are made from a 2 per cent. solution, but another method is to apply a layer of dry permanganate for 15 minutes. A scab forms and leaves a healthy wound which soon heals completely. E. Swales [16] has carried out with great success in two patients a plan of treatment by X-rays and the administration of urea. The doses commenced with 20 grains three times a day, and were increased up to 120 grains. The rays were applied daily, and an improvement in general health accompanied the cure of the lupus. Ormerod,[17] in discussing the difficulties of diagnosis of L. erythematosus lays stress on the absence of nodules and ulceration, the rarity of attacks on the palms, soles, or mucous membranes, and the fact that it commences usually in middle life and is unknown in early youth, while the affected parts are often symmetrical, and from the scales tags may sometimes be seen dipping down, as is said, into the sebaceous glands. Galloway[18] recently showed a case, however, where the mucous membrane of the mouth was affected, and remarks that this rare condition is more likely to be found in acute cases or those where the disease is spread over a large part of of the body. Saalfeld[19] considers that a connecting link between the two forms of lupus is seen in Lupus follicularis, which, like L. erythematosus, generally begins with seborrhœa, and like L. vulgaris shows granulomatous growths.

**Epithelioma of the Skin.**—Allen[20] recommends curetting the margin and removing any scab at the same time. When the oozing has ceased he covers the raw surface with equal parts by weight of arsenious acid and orthoform made into a paste with water, which gives very little pain. To protect the surrounding skin he paints on a ring of collodion coloured with methylene blue. A dressing is placed over the paste and the edges are sealed down with collodion. The effect should be observed at the end of eight or ten hours, and then continued if there is little reaction even twice that time, or it may be removed at once.

**Rodent Ulcer.**—Dubreuilh and Auché[21] find that cases occur as early as 12 years and as late as 83, and not infrequently there is a history of a local injury. The disease commences as a nodule which, as it grows, presses away the connective tissue so as to form an enclosing sheath. Trabeculæ arise from this sheath which divide up the epithelial growth into lobules. Degenerative changes follow. The starting point of the growth is most often from a pilo-sebaceous follicle, never from a sweat gland. Clinically, two types may be seen, the nodular and the ulcerated. Carle, on the other hand, thinks that sweat glands, sebaceous glands, or any one of the elements of the skin may be the starting-point of the growth.

**Leukoplakia.**—J. V. Shoemaker[22] remarks that this affection must be distinguished from tuberculosis, syphilis, epithelioma, and glass-blowers' patches. It begins as a hyperæmic patch which later on becomes white or grey, as the epithelium becomes thickened. Fissures may arise, and sometimes an epithelioma may develop from it. It is not a syphilitic disorder, for patients suffering from it may contract syphilis later on. It occurs not only on the tongue, but also in the larynx, nose, middle ear, vulva, and even in the bladder. The question has been much discussed whether it is identical with psoriasis of the skin. A pigment formed of corrosive sublimate and chromic acid, each of 1 in 200 strength, or a 10 per cent. solution of lactic acid, or a rather stronger solution of salicylic acid have been found useful in treatment, and success has been obtained by decortication of the spot by the knife.

[10] Brit. Med. Jour., Jan. 4. [11] Lancet, Feb. 1. [12] Lancet, March 29. [13] Amer. Jour. Med. Sci., March, p. 557. [14] Brit. Med. Jour., Feb. 22. [15] Lancet, Nov. 9. [16] Lancet, March. [17] Clin. Jour., Jan. 15. [18] Clin. Jour., Dec. 11. [19] Treatment, Jan. [20] Edin. Med. Jour., April. [21] Brit. Jour. Dermat., April, p. 180. [22] Brit. Jour. Dermat., April, p. 154.

